# Knowledge, Attitudes and Intentions to Prescribe Antibiotics: A Structural Equation Modeling Study of Primary Care Institutions in Hubei, China

**DOI:** 10.3390/ijerph16132385

**Published:** 2019-07-05

**Authors:** Chenxi Liu, Chaojie Liu, Dan Wang, Xinping Zhang

**Affiliations:** 1 School of Medicine and Health Management, Tongji Medical School, Huazhong University of Science and Technology, Wuhan 430030, China; 2 School of Psychology and Public Health, La Trobe University, Melbourne, Victoria 3083, Australia

**Keywords:** antibiotics, prescribing behavior, Knowledge-Attitude-Practice, primary care, structural equation modelling

## Abstract

The aim of this paper is to measure the knowledge and attitudes of primary care physicians toward antibiotic prescriptions and their impacts on antibiotic prescribing. A questionnaire survey was conducted on 625 physicians from 67 primary care facilities in Hubei, China. Structural equation modelling (SEM) was applied to test the theoretical framework derived from the Knowledge, Attitudes, and Practices (KAP) theory. Physicians’ knowledge, five sub-types of attitudes, and three sub-types of behavioral intentions towards antibiotic use were measured. Physicians had limited knowledge about antibiotic prescriptions (average 54.55% correct answers to 11 questions). Although they were generally concerned about antibiotic resistance (mean = 1.28, SD = 0.43), and were reluctant to be submissive to pressures from consumer demands for antibiotics (mean = 1.29, SD = 0.65) and the requirements of defensive practice (mean = 1.11, SD = 0.63), there was a lack of motivation to change prescribing practices (mean = −0.29, SD = 0.70) and strong agreement that other stakeholders should take the responsibility (mean = −1.15, SD = 0.45). The SEM results showed that poor knowledge, unawareness of antibiotic resistance, and limited motivation to change contributed to physicians’ high antibiotics prescriptions (*p* < 0.001). To curb antibiotic over-prescriptions, improving knowledge itself is not enough. The lack of motivation of physicians to change needs to be addressed through a systematic approach.

## 1. Introduction

Antibiotic resistance (AR) has become one of the most serious global issues of concern for health development today, threatening our ability to treat common infectious diseases [1]. AR can not only prolong illness, impose additional medical expenditure, and increase mortality, but also deter some common medical procedures, for example, caesarean sections, due to increased risk of infections [2]. There is a consensus that the misuse and overuse of antibiotics has contributed to the problem of AR [3,4,5]. Inappropriate and over-prescription of antibiotics are prevalent worldwide [6]. It was estimated that, in the USA, 30% of antibiotics are over-prescribed in outpatient settings [7], and the percentage of inappropriate antibiotic prescriptions can be as high as 50% [8,9]. Irrational use of antibiotics is an even more serious problem in developing countries because of their fragile regulation systems and a lack of human capacity [10]. 

Physicians play a critical role in the global campaign against AR, simply because prescriptions are required for antibiotic usage [11,12]. It is essential to understand how physicians prescribe antibiotics [11,13]. Several systematic reviews concluded that both intrinsic factors (such as knowledge and attitudes of physicians) and external factors (such as system and organizational environment) have shaped the antibiotic prescribing behaviors of physicians [11,12,14]. However, our understanding about their underlying mechanisms is still limited. Despite the existence of practice guidelines, physicians may defy the guidelines and prescribe antibiotics in order to meet patient expectations or to avoid potential confrontations and complaints from patients [15,16,17,18]. This process could involve some further compromises given that most prescribers are likely to be aware of the side-effects of the overuse of antibiotics. Adding to the complexity is the impact of contextual factors. Physicians can be incentivized by professional, financial, regulatory, and cultural factors. There is a particular shortage of research documenting how physicians prescribe antibiotics in developing countries [19,20,21,22,23].

China has the largest consumer population for antibiotics in the world. Meanwhile, the overuse of antibiotics is also prevalent in China. Inappropriate and over-prescription of antibiotics are widespread across the entire health industry, whether in hospitals or in primary care facilities. Over half of the vast volume of patient visits (43.67 billion in 2016 [24]) involve a prescription containing antibiotics [10,25,26,27]. However, less than 40% of these antibiotic prescriptions are appropriate [25]. Little is known in regard to the knowledge, attitudes, and behaviors of physicians in China toward antibiotic prescriptions. Therefore, this study aimed to fill in the gap in the literature.

## 2. Participants and Methods

### 2.1. Settings

This study was conducted in Hubei Province of China, with a focus on primary care facilities. In China, about 60% of outpatient visits occur in primary care facilities [24]. Hubei is located in Central China and has a population of 58.85 million. Its socio-economic development ranks in the middle (11/32) of all regions in China [24].

This study covered both urban community health centers (CHCs) and rural township health centers (THCs). There are 342 CHCs and 1139 THCs in Hubei. In 2016, they received 20.44 million and 58.00 million patient visits, respectively [24]. A study revealed that about 60% of primary care visits in Hubei involved an antibiotic prescription [28], which is double the recommended level (30%) from the World Health Organization [29].

### 2.2. Theoretical Framework

**Figure 1:**
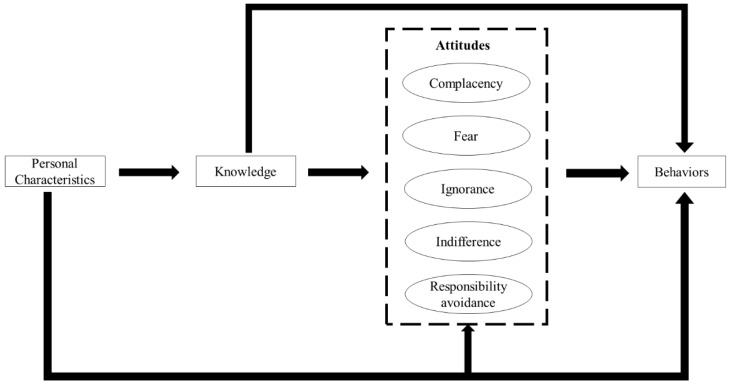
Theoretical framework of knowledge, attitudes, and behaviors in regard to antibiotic prescriptions.

We adapted the theoretical model proposed by Teixeira (Figure 1). The model was developed based on the theory of knowledge, attitudes, and practices (KAP) [30]. We revised the model based on several systematic reviews [11,12,14]. Knowledge was tested using 11 standard questions [31,32,33]. Attitudes were categorized into five aspects [11,12]:

- Complacency: prescribing antibiotics to satisfy patient demands and expectations;
- Fear: prescribing antibiotics for fear of losing patients or losing in potential disputes with patients;
- Ignorance: a lack of concern in relation to antibiotic resistance resulting from over-prescriptions of antibiotics;
- Indifference: a lack of motivation to change antibiotic prescribing practices; and
- Responsibility avoidance: a belief that others (patients, governments and other professionals) are responsible for the problem of antibiotic resistance.

The model hypothesized that knowledge can directly link to prescribing practices. Meanwhile, the impacts of knowledge on antibiotic prescriptions can also be enhanced or jeopardized by the above five aspects of attitudes. In addition, the personal characteristics of prescribers may also exert some influence on certain knowledge, attitudes, and prescribing behaviors (Figure 1).

### 2.3. Survey Instruments

A 54-item questionnaire (Table S1) was developed measuring the knowledge, attitudes, behaviors, and personal characteristics of physicians associated with antibiotic prescriptions.

Knowledge was measured using 11 questions, asking the respondents to make a judgment on the circumstances in which antibiotics (or a type of antibiotic) should or should not be prescribed. Eight of these questions were borrowed from previous studies conducted in Peru, DR Congo, and Laos [31,32,33]. We added three additional questions: two in relation to antibiotic prescriptions for upper respiratory tract infections (URTIs) [34,35] because inappropriate use of antibiotics for URTIs has remained a serious problem in China [25]; and one about the WHO recommendations for antibiotic use in primary care [29]. Each question contained four or five alternative answers, with only one being deemed correct. The respondents were also given a chance to choose “unknown” to discourage guessing. 

Attitudes were measured using 27 items along a five-point Likert scale, with each subscale containing a minimum of three items. These items were adapted from two validated instruments [36,37], taking into consideration the findings from several literature reviews [11,12,14] and an exploratory study on prescribing practices in primary care in China [38]. 

Intentions to prescribe antibiotics were measured in this study as a proxy indicator for prescribing practices. Such a strategy has been successfully applied in previous studies [39,40,41,42]. Empirical evidence shows that behavioral intentions can predict about 20–30% of actual behaviors [43] and they are more sensitive to changes when actual behaviors are not readily observed [44]. In this study, three subscales were included: intentions to prescribe antibiotics; intentions to reduce antibiotic prescriptions; and intentions to prescribe antibiotics for URTIs. The first two subscales were measured using three items each, tapping into the actions of “want, expect, and plan”, respectively, along a five-point Likert scale, whereas the last one used a single item estimating the number of URTI patients (out of 10) that would be prescribed with antibiotics. This question was designed to capture the most prominent challenge in China [25]. 

The personal characteristics captured in this study included age, gender, education, income, medical sub-department they worked at, job title, years of practice, and training in relation to antibiotics. 

The development and modification of the questionnaire followed the guidelines of the KAP survey [30] and the validation procedure of survey instruments [45,46] (see Figure S1 for details of the questionnaire development). A double translation (forward-translation and backward-translation) process was applied to ensure consistency between the original English questionnaire and the translated Chinese version. A pilot study was conducted on 21 physicians from three primary care facilities. The participants were asked to complete the questionnaire and provide feedback about the relevance, clarity and difficulty of the questionnaire items. This led to the revision, addition, or removal of some items. The validity and reliability of the Chinese questionnaire were confirmed in the final survey (*n* = 625) through confirmatory factor analysis (CFA) and Cronbach’s alpha. The CFA results demonstrated an excellent fitness of data into the hypothetical model [47,48]: root mean square error of approximation (RMSEA) = 0.047 (<0.08); Tucker–Lewis index (TLI) = 0.980 (>0.95); comparative fit index (CFI) = 0.977 (>0.95). High internal consistency was evident as indicated by the high Cronbach’s alpha for the sub-domains of attitudes (α = 0.669–0.912) and behaviors (α = 0.893–0.898), except for the attitude “responsibility avoidance” (α = 0.385). We presented the descriptive results of “responsibility avoidance” given that it was reported in previous studies [37]. However, it was excluded from further analyses in the modelling. 

### 2.4. Sampling and Data Collection

A stratified cluster random sampling strategy was adopted. Hubei Province is geographically divided into western, central, and eastern regions. We estimated that at least 17 clusters with a sample size of 167 responses would be required in each region using the sample size calculator developed by Dhand and Khatkar (with an expected deviation < 6, precision = 1, level of confidence = 95%, inter-class correlation coefficient < 0.02 and cluster size = 10) [49]. In each region, one urban city and two rural counties were randomly selected. Then, eight primary care facilities (urban CHCs or rural THCs) were randomly selected (or all primary care facilities if less than eight) in each selected city or county. All of the physicians on duty over the period of the survey were approached and invited to participate in the survey. 

**Figure 2:**
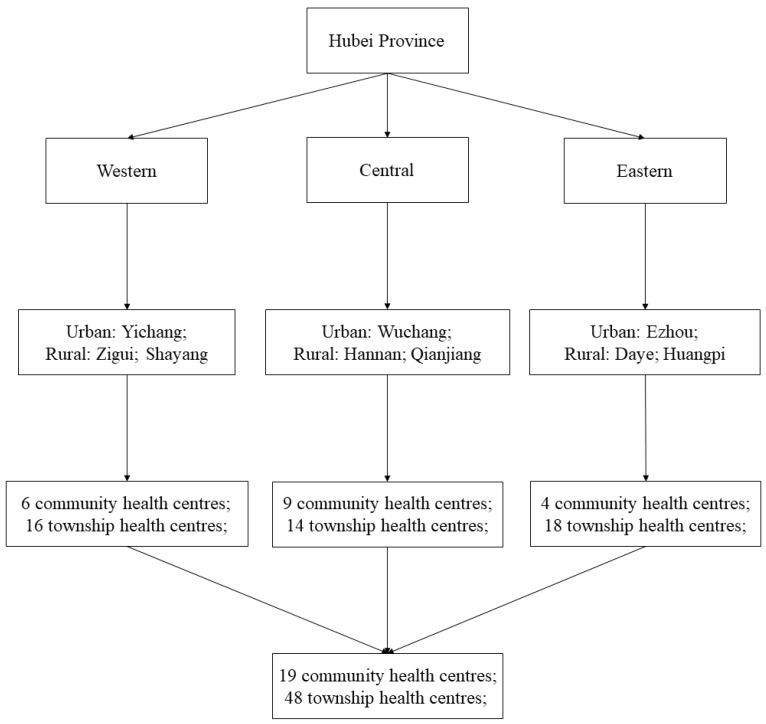
Sampling procedures.

If less than 70 physicians were identified from a city or county, an additional primary care facility (if available) was added to the sample. This resulted in a final sample of 67 primary care facilities, including 19 urban CHCs and 48 rural THCs (Figure 2). 

Data were collected over the period from 23 April to 6 June in 2018. Each facility was visited by a pair of trained investigators (recruited from postgraduate research students in social sciences and medicine). The recruited investigators have learned theories and methods of social investigation from their postgraduate courses and received a one-day intensive training, covering the background of the current survey, detailed interpretation of survey instrument, and a simulation survey test. Finally, a total of 10 investigators were recruited. They approached all of the physicians on duty, but only those who prescribed antibiotics were invited to participate in the survey. The physicians working in the administrative departments and those whose tasks rarely involved antibiotic prescriptions (such as exclusive duties on maternal care and vaccinations) were excluded from the survey. The investigators explained the purpose and procedure of the study and obtained written informed consent from each respondent before the respondent was asked to self-complete the questionnaire. On average, the survey took 10–15 min to complete. The completeness of the returned questionnaire was examined by the investigators, with missing items (if existing) being amended through complementary interviews on the spot. A token gift (roughly $1.65) was given to the participant upon completion of the survey. 

In total, 712 questionnaires were distributed and 664 were returned. Of the returned questionnaires, 625 contained no missing items and were included for further analyses. This represented an effective response rate of 87.78%. 

### 2.5. Data Analysis

Knowledge about antibiotic prescriptions was assessed using 11 questions. The percentage of respondents giving a correct answer to each question and the total number of correct answers per respondent were calculated. 

Each attitude item was coded along a five-point Likert scale, with a negative score indicating disagreement and a positive score indicating agreement with the relevant evidence-informed good practice. The scores in the same sub-domain were added and averaged (ranging from −2 to 2). 

Prescribing behavioral intentions were assessed using three indicators: percentage of prescriptions containing antibiotics for URTIs, average score for the efforts to prescribe antibiotics, and average score for the efforts to reduce antibiotic prescriptions. The latter two were coded in a similar way as the attitude measurements (see details about the responses to attitude and behavioral items in Table S2), with a negative score indicating refusal and a positive score indicating supportive of reducing antibiotic prescriptions (ranging from −2 to 2). 

The differences between the respondents in their knowledge, attitudes, and practices toward antibiotic prescriptions were examined using chi-square (or Fisher’s exact tests), Kruskal–Wallis rank tests, or one-way analysis of variance (ANOVA). 

**Figure 3:**
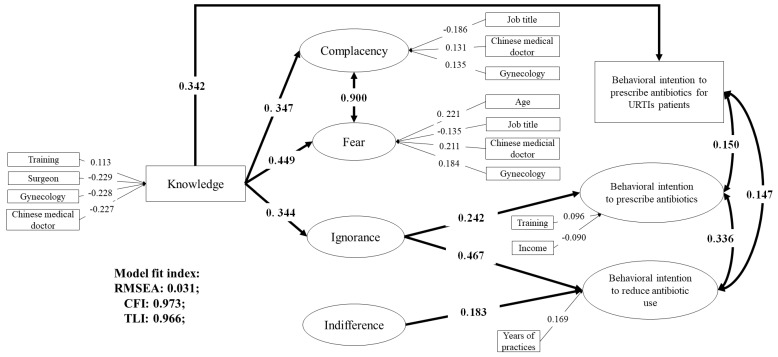
Structure equation model on knowledge, attitudes, and behavioral intentions toward antibiotic prescriptions. Only significant pathways (*p* < 0.05) were reported with standardized path coefficients.

Structural equation modelling (SEM) was applied to establish the associations between knowledge, attitudes and practices (Figure 1). Means and variance adjusted weighted least squares (WLSMV) estimation was adopted in the SEM, which was designed for ordinal data (e.g., five-point Likert scale) [50]. We used a mixed-model, adjusting for the cluster effect (at the facility level). Figure 3 presents the results of standardized path coefficients with statistical significance (*p* < 0.05). The fitness of data into the SEM model was assessed using several recommended criteria [47,48]: RMSEA < 0.08; TLI > 0.95; and CFI > 0.95. Modifications on the original hypothetical model were made based on the modification index. 

The statistical analyses were performed using STATA (version 12.0) (StataCorp., College Station, TX, USA) and Mplus (version 6.0) (Muthén & Muthén, Los Angeles, CA, USA). A *p*-value < 0.05 was considered statistically significant.

## 3. Results

### 3.1. Characteristics of Respondents

The 625 respondents had a mean age of 43.27 years (standard deviation, SD = 10.43) and most (69.76%) were male. The majority (78.08%) of respondents came from rural THCs. On average, the respondents had 16 years of clinical experience and over three quarters (76.32%) had received training in relation to antibiotics over the last year prior to the survey (Table 1). 

### 3.2. Knowledge, Attitudes, and Behavioral Intentions Toward Antibiotic Prescriptions

On average, the respondents answered six questions correctly (SD = 1.46) of a total of 11 (Table 2). Incorrect answers were most likely to appear in antibiotic prescriptions for URTIs (94.24%), followed by dosage adjustment of antibiotics for renal failure (89.76%), and effective antibiotic treatment for methicillin-resistant Staphylococcus aureus (70.88%). About 60% of respondents could not determine the antibiotics that most effectively crosses the blood-brain barrier. On average, general practitioners and internists/pediatricians had higher scores for antibiotic knowledge than their other sub-specialized colleagues (*p* < 0.001).

On average, the respondents reported a positive attitude toward rational antibiotic prescriptions in response to pressures from patient expectations (complacency score = 1.29, SD = 0.65) and the requirements of defensive practice (fear score = 1.11, SD = 0.63). There was a relatively high level of concern about antibiotic resistance resulting from over-prescriptions (ignorance score = 1.28, SD = 0.43). However, a shortage of motivation in changing antibiotic prescribing practices was evident: a negative score (−0.29) was shown in indifference (SD = 0.70). The respondents were inclined to believe that the solution to antibiotic resistance went beyond their own responsibilities (responsibility avoidance score = −1.15, SD = 0.45) (Table 3). 

The respondents reported that they would prescribe antibiotics to about 40% (SD = 22%) of patients with URTIs. However, a relatively strong intention to reduce antibiotic prescriptions (mean score = 1.29, SD = 0.54) was reported, compared with the intention to prescribe antibiotics (mean score = 0.86, SD = 0.63).

The attitudes and behavioral intentions toward antibiotic prescriptions were consistent across different subspecialties, except for the gynecologists and Chinese medical practitioners who reported less pressure of defensive practice (fear) compared with other sub-specialties (*p* = 0.002).

### 3.3. Associations between Knowledge, Attitudes, and Behavioral Intentions

The SEM confirmed the theoretical framework for the antibiotic prescribing behaviors of physicians with some modifications (Figure 3). The final model had a good fitness of data: RMSEA = 0.031 (95% CI: 0.028–0.034), CFI = 0.973, and TLI = 0.966). 

Overall, a high level of knowledge was associated with a more positive attitude and behavioral intention for containing antibiotic prescriptions. Higher knowledge scores were found to be linked with less complacency (β = 0.347, *p* < 0.001), less fear (β = 0.449, *p* < 0.001), and less ignorance (β = 0.344, *p* < 0.001), but not less indifference. Knowledge had indirect effects on intentions to prescribe antibiotics through the attitude of ignorance. However, the attitudes of complacency and fear were not linked to intentions to prescribe antibiotics (*p* > 0.05). Poor antibiotic knowledge was also directly linked to the intention to prescribe antibiotics for URTIs (β = 0.342, *p* < 0.001).

A high level of concern about antibiotic resistance (attitude of ignorance) was linked with low intentions to prescribe antibiotics (β = 0.242, *p* < 0.001) and high intentions to reduce antibiotic prescriptions (β = 0.467, *p* < 0.001). Intentions to reduce antibiotic prescriptions were also associated with the motivation of behavioral changes (attitude of indifference: β = 0.183, *p* < 0.001).

The characteristics of respondents were associated with their knowledge, attitudes, and behavioral intentions toward antibiotic prescriptions. Compared with general practitioners, surgeons (β = −0.229, *p* < 0.001), gynecologists (β = −0.228, *p* = 0.001) and Chinese medical doctors (β = −0.227, *p* < 0.001) had poorer knowledge about antibiotic prescriptions; but the latter two were less submissive to pressures from patient demands and defensive practice (Figure 3). 

Antibiotic training appeared to be associated with better knowledge (β = 0.113, *p* = 0.034) and lower intentions (β = 0.096, *p* = 0.027) to prescribe antibiotics. Higher income was associated with higher intentions to prescribe antibiotics (β = −0.090, *p* = 0.045). Increased years of clinical practices was associated with higher intentions to reduce antibiotic prescriptions (β = 0.169, *p* = 0.005). 

## 4. Discussion

This study confirmed the theoretical framework based on the KAP model, indicating that the intention of physicians to reduce antibiotic prescriptions is shaped by relevant knowledge and attitudes. The results show that although knowledge plays an important role in shaping how physicians respond to different contextual factors originating from patients, colleagues, and managers, these responses are not always associated with intentions to prescribe (or not to prescribe) antibiotics. Better knowledge may ease the pressure of antibiotic prescriptions resulting from patient demands or defensive practice. However, this may not eventually result in a reduction of antibiotic prescriptions. On the other hand, knowledge bears no connection with the motivations of physicians for behavioral changes, in spite of the importance of motivations for changing prescribing practices. One potential pathway for knowledge to play a role in changing prescribing practices, however, is to promote the acknowledgement of the importance of prescribing practices in curtailing antibiotic resistance. 

### 4.1. Knowledge

This study revealed low levels of antibiotic knowledge in the study participants. The respondents scored on average 55% of correct answers about antibiotic prescriptions (6.04 out of 11 questions), compared with 60–86% for physicians in hospitals from Lao, DR Congo, and Peru. This knowledge gap is even larger in antibiotic treatments for URTIs: 5% versus 35–76% [31,32,33,34]. The SEM indicates that low knowledge is directly linked to intentions to prescribe antibiotics for URTIs. The respondents in this study reported that they would prescribe antibiotics for 40% of patients with URTIs, which is not recommended in the practice guidelines in the USA and the UK [51,52]. In reality, the over-prescription of antibiotics for URTIs may be even more serious. Empirical evidence shows that 80% of outpatient visits for URTIs in China involved an antibiotic prescription, for which 80% are inappropriate [25]. 

Training can contribute to knowledge acquisition, as indicated in the SEM results. However, the current training programs may have contributed little, if any, to the improvement of the antibiotic knowledge of physicians. Over three-quarters of respondents in this study reported experience of training in relation to antibiotics. However, their antibiotic knowledge level remained low. Since 2009, China has made great efforts in training primary care workers [53]. However, the training programs have largely been theoretically driven with a shortage of consideration of incentives and motivations [53]. There is also a lack of detailed guidelines about how to educate prescribers in the “Guiding Principles for Clinical Application of Antimicrobial Agents” published in 2015 in China [54]. Some researchers expressed concerns about some outdated recommendations included in the guiding principles. For example, antibiotics are recommended for patients with purulent rhinitis, which has no, or at best limited, benefits [51,52,55]. 

### 4.2. Attitudes

Attitudes can undermine practices. We found that our study participants reported highly positive attitudes in dealing with pressures from consumer demands for antibiotics and the requirements of defensive practice. However, these attitudes are not connected with intentions to prescribe (or not to prescribe) antibiotics. A survey in Shandong revealed a similar result, in which 88% of physicians would refuse to prescribe antibiotics they considered unnecessary even when patients insisted on it [56]. However, several studies conducted in the UK, the USA, the Netherlands, and Australia showed that complacency and fear may be main drivers for physicians to prescribe antibiotics [15,16,17,57,58], which is inconsistent with what we found in this study. It is important to note that China already has a very high level of antibiotic prescriptions embedded in the culture of practices [10,25,26,27]. In addition, unlike primary care physicians in some other countries [12,15,16,17,57,58], further compromise with patient demands would not give them any additional benefits given that primary care workers were fully salaried by the governments [59] and their incomes were decoupled with services they provided [60]. 

The attitudes of ignorance and indifference were found to be associated with antibiotic prescribing intentions. The study participants had already demonstrated quite a positive attitude toward acknowledging the negative consequences (antibiotic resistance) of over-prescriptions, although it may be further strengthened through better knowledge. Therefore, the greatest challenge may lie in the lack of motivation of physicians to change practices as indicated by the negative average score in the attitude of indifference. Indifference may lead to low intentions to reduce antibiotic prescriptions. The current high level of antibiotic prescriptions is likely to continue if the motivation issue is left unaddressed, especially when high workloads are common in physicians [61]. However, knowledge improvement does not offer a solution to the issue because it has no impact on the attitudes of indifference.

It is a serious issue of concern that primary care physicians may find excuses for not taking responsibility themselves in fighting antibiotic resistance. There was an overwhelming belief by the study participants that other people are responsible for antibiotic resistance. However, empirical evidence shows that antibiotic prescribing in primary care has contributed significantly to the development of antibiotic resistance [3]. China is not alone. Such a “not in my backyard” attitude is prevalent worldwide [14] and can even lead to increased antibiotic prescriptions [37,62]. 

### 4.3. Policy Implications

The campaign for reducing over-prescriptions of antibiotics should take a systems approach, addressing problems associated with both the knowledge and attitudes of prescribers. Training programs and practice guidelines should target major gaps in the knowledge of prescribers (e.g., antibiotic prescription for URTIs), involving not only general practitioners but also other sub-specialists, such as surgeons and gynecologists [63,64]. Greater efforts need to be made to motivate physicians to change their prescribing behaviors. There is emerging evidence to show that increased transparency and public reporting may work in favor of curtailing over-prescriptions of antibiotics [28,65]. Governments, professional bodies, and consumers should all play a role in the campaign. In the public sector, governmental funding should reward good practices [66], which has been proven to be an effective approach for reducing antibiotic prescriptions. Medical professional bodies should promote a high level of professionalism through strong codes of conduct [67] and multi-disciplinary collaborations [68]. Improved health literacy and engagement of consumers can also help foster a better clinical environment for prescribers [69]. 

### 4.4. Strengths and Limitations

This study adopted a SEM approach to test the theoretical framework based on KAP, which enabled us to explore multiple factors associated with antibiotic prescriptions [11]. This fills a significant gap in the literature documenting antibiotic prescriptions in China [10,38,56]. The study used a validated instrument that had been absent in this field [70].

There are several limitations in this study: (1) The study was conducted in primary care facilities in one province (Hubei) of China. Attempts to generalize findings of this study should be cautious. (2) We measured behavioral intentions instead of practices. This may result in an overestimation of the tested effects since intentions do not always translate into practices. (3) Responsibility avoidance, an important construct of attitudes, was not included in the SEM simply because of its low Cronbach’s α. Further studies are warranted to explore the underlying reasons. Some researchers recommend separate measurements of attitudes toward the responsibilities of different stakeholders (e.g., patients, governments, pharmacists, and others) [36,37]. (4) Though we contacted each surveyed facility in advance and approached physicians at their preferred time, to ensure that they have sufficient time to carefully reply to the questionnaire, the survey context could be difficult to control and it should be noticed that the variability in responses could be partly due to variability in the time/method of data collection, since physicians may be more inclined to skim rather than read the questionnaire carefully during busy times. (5) Thirty-nine questionnaires were excluded due to large numbers of unfilled items and the respondents refused to finish them when investigators asked whether they could complete them based on the immediate check when respondents returned their reply. It is possible that the omitted 39 questionnaires may have introduced bias. However, since the omitted questionnaires were excluded and not imported into the dataset of current study. We were not able to assess whether the answers of the excluded questionnaires were similar to the included 625 questionnaires. In addition, since items of the excluded questionnaires were largely missed, it may be difficult to conduct such analyses. Considering the response rate is 87.78%, the included 625 responses may be well-representative of the physicians in primary care facilities and the inclusion of omitted responses may limitedly change the results of the current study. 

## 5. Conclusions

Physicians in primary care facilities in Hubei have low levels of knowledge about antibiotic prescriptions. This is connected with a high level of antibiotic prescriptions for URTIs in particular. These physicians are concerned about antibiotic resistance resulting from over-prescriptions of antibiotics. Generally speaking, they are reluctant to be submissive to pressures from consumer demands for antibiotics and the requirements of defensive practice. However, there is a lack of motivation to change prescribing practices although high levels of antibiotic prescriptions are evident in China. There is a tendency to shift responsibilities to other stakeholders. Improving knowledge may lead to higher motivation and result in fewer antibiotic prescriptions. However, responsibility avoidance can be a serious barrier for mobilizing health professionals, which should be addressed through a broad systems approach that goes beyond training and practice guidelines.

## Figures and Tables

**Table 1:** Characteristics of respondents.

Characteristics	Mean ± SD */*N* (%)
Age (years)	43.27 ± 10.43
Gender	
Male	436 (69.76)
Female	189 (30.24)
Facility	
Urban community health center	137 (21.92)
Rural township health center	488 (78.08)
Medical sub-specialization	
General practitioner	264 (42.24)
Internist/pediatrician	154 (24.64)
Surgeon	77 (12.32)
Gynecologist	87 (13.92)
Chinese medical practitioner	43 (6.88)
Professional title	
Junior doctor	324 (51.84)
Attending doctor	236 (37.76)
Associate senior or senior consultant	65 (10.40)
Level of education	
Vocational training	51 (8.16)
Associate degree	329 (52.64)
University degree	245 (39.20)
Annual household income (Chinese RMB ¥)	
<40,000	169 (27.04)
40,000~	305 (48.80)
80,000~	107 (17.12)
≥120,000	44 (7.04)
Clinical experience (years)	16.64 ± 11.11
Training about antibiotics over the last year	
Yes	477 (76.32)
No/Not aware	148 (23.68)

* SD: Standard Deviation.

**Table 2:** Knowledge of respondents about antibiotic prescriptions.

Knowledge Questions	Number (Percentage) of Respondents Giving a Correct Answer						*p* *-Value
	Total *n* = 625	General Practitioner *n* = 264	Internist/Pediatrician *n* = 154	Surgeon *n* = 77	Gynecologist *n* = 87	Chinese Medical Practitioner *n* = 43	
Antibiotics should not be prescribed for non-febrile diarrhea	591 (94.56)	253 (95.83)	149 (96.75)	70 (90.91)	84 (96.55)	39 (90.70)	0.171
Antibiotics should not be prescribed for upper respiratory tract infections	36 (5.76)	15 (5.68)	9 (5.84)	5 (6.49)	5 (5.75)	2 (4.65)	0.998
Dosage reduction of antibiotics is needed for renal failure	64 (10.24)	21 (7.95)	11 (7.14)	12 (15.58)	17 (19.54)	3 (6.98)	0.011
Amoxicillin is a safe antibiotic product for pregnant patients	596 (95.36)	254 (96.21)	148 (96.10)	72 (93.51)	87 (100.00)	35 (81.40)	<0.001
Metronidazole has the best activity against anaerobes	601 (96.16)	261 (98.86)	150 (97.40)	73 (94.81)	80 (91.95)	37 (86.05)	<0.001
Methicillin resistant staphylococcus aureus is resistant to beta- lactam antibiotics	182 (29.12)	86 (32.58)	49 (31.81)	13 (16.88)	19 (21.84)	15 (34.88)	0.027
Ceftriaxone most effectively crosses the blood-brain barrier	246 (39.36)	120 (45.45)	53 (34.41)	27 (35.07)	33 (37.93)	13 (30.23)	0.102
Aminoglycosides are very active if they are administered as parenteral once daily	286 (45.76)	126 (47.73)	66 (42.85)	36 (46.75)	43 (49.43)	15 (34.88)	0.483
Bacterial pneumonia (including one of the following symptoms: fast breathing, chest in-drawing or stridor) requires antibiotic treatment	311 (49.76)	145 (54.92)	83 (53.89)	33 (42.86)	33 (37.93)	17 (39.53)	0.017
Antibiotics do not reduce the duration and the occurrence of complications of upper respiratory tract infections	380 (60.80)	177 (67.05)	113 (73.37)	35 (45.45)	36 (41.38)	19 (44.19)	<0.001
The average number of patients taking antibiotics should be below 30 per 100 in a primary care facility	478 (76.48)	218 (82.58)	119 (77.27)	51 (66.23)	57 (65.52)	33 (76.74)	<0.001
Overall score (mean ± SD)	6.04 ± 1.46	6.34 ± 1.36	6.16 ± 1.43	5.55 ± 1.53	5.68 ± 1.34	5.30 ± 1.70	<0.001

* *p*-values derived from Fisher’s exact tests or one-way analysis of variance.

**Table 3:** Attitudes and behavioral intentions of respondents toward antibiotic prescriptions.

Measurement	Scores (Mean ± SD)						*p* *	Cronbach’s Alpha
	Total *n* = 625	General Practitioner *n* = 264	Internist/Pediatrician *n* = 154	Surgeon *n* = 77	Gynecologist *n* = 8 7	Chinese Medical Practitioner *n* = 43		
Attitude								
Complacency	1.29 ± 0.65	1.26 ± 0.65	1.30 ± 0.68	1.22 ± 0.69	1.41 ± 0.56	1.36 ± 0.69	0.173	0.912
Fear	1.11 ± 0.63	1.07 ± 0.64	1.09 ± 0.64	1.00 ± 0.62	1.31 ± 0.54	1.27 ± 0.67	0.002	0.797
Ignorance	1.28 ± 0.43	1.32 ± 0.44	1.24 ± 0.42	1.21 ± 0.41	1.26 ± 0.38	1.27 ± 0.57	0.140	0.694
Indifference	−0.29 ± 0.70	−0.29 ± 0.70	−0.27 ± 0.67	−0.32 ± 0.77	−0.36 ± 0.64	−0.22 ± 0.74	0.775	0.669
Responsibility avoidance	−1.15 ± 0.45	−1.22 ± 0.45	−1.19 ± 0.46	−1.15 ± 0.46	−1.14 ± 0.39	−1.17 ± 0.48	0.286	0.385
Behavioral intention								
Prescribe antibiotics for upper respiratory tract infections	3.98 ± 2.21	3.94 ± 2.09	3.86 ± 2.28	4.58 ± 2.57	3.92 ± 2.14	3.65 ± 2.02	0.221	N/A
Prescribe antibiotics	0.86 ± 0.63	0.84 ± 0.61	0.83 ± 0.62	0.86 ± 0.73	0.95 ± 0.59	0.86 ± 0.67	0.761	0.898
Reduce antibiotic prescriptions	1.29 ± 0.54	1.31 ± 0.52	1.24 ± 0.55	1.30 ± 0.54	1.36 ± 0.50	1.22 ± 0.64	0.694	0.893

* *p*-values derived from Kruskal–Wallis rank tests; N/A: Not applicable.

## Supplementary Materials

The following are available online at https://www.mdpi.com/1660-4601/16/13/2385/s1; Figure S1. Development of the survey questionnaire; Table S1. Survey instruments (translated version); Table S2. Physicians’ responses to individual items.

## List of Abbreviations

SEM: Structural equation modelling; KAP: Knowledge, Attitudes and Practices; AR: Antibiotic resistance; CHCs: Community health centers; THCs: Township health centers; URTIs: Upper respiratory tract infections; CFA: Confirmatory factor analysis; RMSEA: Root mean square error of approximation; TLI: Tucker–Lewis index; CFI: Comparative fit index; ANOVA: One-way analysis of variance; WLSMV: Means and variance adjusted weighted least squares; SD: Standard deviation.
